# Conjugation of Inulin Improves Anti-Biofilm Activity of Chitosan

**DOI:** 10.3390/md16050151

**Published:** 2018-05-04

**Authors:** Guiqiang Zhang, Jing Liu, Ruilian Li, Siming Jiao, Cui Feng, Zhuo A. Wang, Yuguang Du

**Affiliations:** 1 University of Chinese Academy of Sciences, Beijing 100049, China; gqzhang@ipe.ac.cn (G.Z.); rlli@ipe.ac.cn (R.L.); 2 Key Laboratory of Biopharmaceutical Production & Formulation Engineering, PLA and State Key Laboratory of Biochemical Engineering, Institute of Process Engineering, Chinese Academy of Sciences, Beijing 100190, China; jingliu0223@126.com (J.L.); smjiao@ipe.ac.cn (S.J.); cfeng@ipe.ac.cn (C.F.); 3 College of Chemistry & Pharmacy, Northwest A&F University, Yangling 712100, China

**Keywords:** *Staphylococcus aureus*, biofilm, chitosan, conjugation, inulin

## Abstract

Bacteria biofilm helps bacteria prevent phagocytosis during infection and increase resistance to antibiotics. *Staphylococcus aureus* is a Gram-positive pathogenic bacterium and is tightly associated with biofilm-related infections, which have led to great threat to human health. Chitosan, the only cationic polysaccharide in nature, has been demonstrated to have antimicrobial and anti-biofilm activities, which, however, require a relative high dosage of chitosan. Moreover, poor water solubility further restricts its applications on anti-infection therapy. Inulins are a group of polysaccharides produced by many types of plants, and are widely used in processed foods. Compared to chitosan, inulin is very soluble in water and possesses a mild antibacterial activity against certain pathogenic bacteria. In order to develop an effective strategy to treat biofilm-related infections, we introduce a method by covalent conjugation of inulin to chitosan. The physicochemical characterization of the inulin–chitosan conjugate was assayed, and the anti-biofilm activity was evaluated against *S. aureus* biofilm. The results indicated that, as compared to chitosan, this novel polysaccharide–polysaccharide conjugate significantly enhanced activities against *S. aureus* either in a biofilm or planktonic state. Of note, the conjugate also showed a broad spectrum anti-biofilm activity on different bacteria strains and low cellular toxicity to mammalian cells. These results suggested that chitosan conjugation of inulin was a viable strategy for treatment against biofilm-related infections. This finding may further spread the application of natural polysaccharides on treatments of infectious disease.

## 1. Introduction

Planktonic bacteria are tightly associated with acute infections and widely used as a target in the development of antibiotics. However, biofilm has been characterized as the dominant living-state of bacteria in nature, and is capable of causing a wide range of chronic, relapsing infectious disease [1]. Biofilms have been defined as a structured community of bacterial cells enclosed in a self-produced matrix of primarily polysaccharide material that is adherent to each other or a surface [2,3]. The matrix is important because it provides structural stability and protection for the bacteria against adverse environmental challenges, including pH extremes, heat, lack of nutrients, and antimicrobial therapy [4,5]. During host infection, forming biofilms help bacteria escape from the attack of the host innate immune system by reducing the effectiveness of antimicrobial peptides and phagocytosis [4,6]. It was reported that bacteria within biofilms were 10–1000 times more resistant to the action of antimicrobials than planktonic bacteria [4]. Until now, drugs specifically targeting bacteria in the biofilm were lacking. Developing effective strategies to prevent and eradicate biofilm infections is urged.

Chitosan, the *N*-deacetylated derivative of chitin, is a linear polysaccharide which composed of d-glucosamine and *N*-acetyl-d-glucosamine units linked by β-1,4-glycosidic linkages [7]. Studies have showed that chitosan has antimicrobial activities against a wide range of microorganisms, such as bacteria, algae, and fungi [8,9,10]. However, the actual inhibitory mechanism has not been fully defined. One possibility is that chitosan interferes with cell permeability, due to interactions between the positively charged polysaccharide and the negatively charged plasma membrane [11]. The anti-biofilm activity of chitosan and its derivatives on bacteria and fungi have also been demonstrated [12,13,14]. However, a relative high dosage of chitosan is usually required to get optimal antimicrobial activity, especially on bacteria in biofilms. Furthermore, the low water solubility and high viscosity also restricts its applications in treating infections. Chemical modification of chitosan has provided a feasible way to overcome these problems to some extent [15,16]. Several studies have showed that modifications by introducing small functional groups to chitosan backbone can increase the solubility of chitosan and improve its bioactivities [17,18].

Different from small molecules, inulin is a water-soluble polysaccharide possessing low toxicity and substantial biocompatibility [19]. Besides, inulin has showed inhibitory activity on the growth of pathogenic bacteria or fungi [20,21]. In this study, the enhanced anti-biofilm activities of chitosan by inulin conjugation were investigated for the development of a high-efficient antibacterial substance with low side effects. To this aim, we covalently linked inulin to chitosan, followed by the assessment of physicochemical characterization of newly-prepared inulin–chitosan conjugate. Furthermore, we evaluated the effect of the inulin–chitosan conjugate on biofilm inhibition, biofilm eradication and activity against planktonic bacteria. Additionally, the broad spectrum anti-biofilm activity and cellular toxicity of the conjugate was also assessed.

## 2. Results

### 2.1. Screening of Chitosan/Chitooligosaccharides (COS)

**Figure 1:**
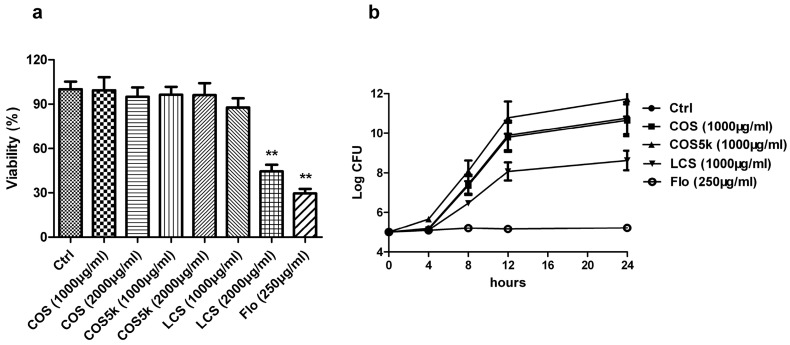
The activities of chitosan/chitooligosaccharide (COS) with different molecular weights against mature *Staphylococcus aureus* biofilm (**a**) and plankton (**b**) were investigated. Florfenicol (250 μg/mL) was used as a positive control. The activity of chitosan/COS samples against mature *S. aureus* biofilm was determined by 3-(4,5-dimethylthiazol-2-yl)-2,5-diphenyl tetrazolium bromide (MTT) assay (**a**). The activity of chitosan/COS (1 mg/mL) samples against *S. aureus* plankton was measured by the method described in 4.7 (**b**). Data are represented as the means ± SD (*n* = 3). ** *p* < 0.01, compared to the control.

To assess the molecular weight of chitosan/chitooligosaccharides on their antibacterial activities, mature *Staphylococcus aureus* biofilm and plankton were treated with chitosan oligosaccharide (<1 kDa, COS), chitosan oligosaccharide (4–6 kDa, COS5k), and low molecular weight chitosan (50–190 kDa, LCS) respectively. Florfenicol (Flo) was used as a positive control. At 2000 μg/mL, LCS showed a strong activity on destructing *S. aureus* biofilm at a similar level as 250 μg/mL florfenicol, while at 1000 μg/mL LCS treatment barely affected biofilm (Figure 1a). COS or COS5k did not show obvious activity at either concentration (Figure 1a). As shown in Figure 1b, LCS inhibited the growth of planktonic *S. aureus* at a concentration of 1000 μg/mL, while COS and COS5k only showed a mild effect at the same concentration. The same trend was showed at a concentration of 2000 μg/mL of each chitosan/chitooligosaccharide samples (Supplementary Figure S2). All these results revealed that LCS exhibited the best activity against biofilms and plankton. 

### 2.2. Preparation and Characterization of the Inulin–LCS Conjugate

**Figure 2:**
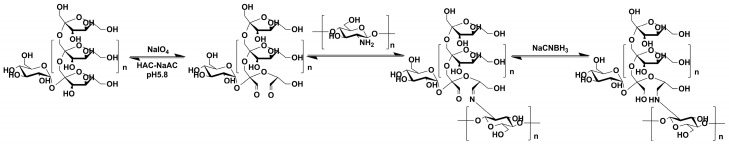
Reaction scheme of the inulin– low molecular weight chitosan (LCS) conjugate synthesis.

To prepare the inulin–LCS conjugate, the ortho-dihydroxyl groups of the inulin were oxidized to the aldehyde group by NaIO_4_. Then, the conjugation between LCS and inulin was achieved by reduction of the resulting Schiff base formed by free amino groups in LCS and aldehyde groups in inulin (Figure 2), as described [22]. 

#### 2.2.1. HPLC Assay

**Figure 3:**
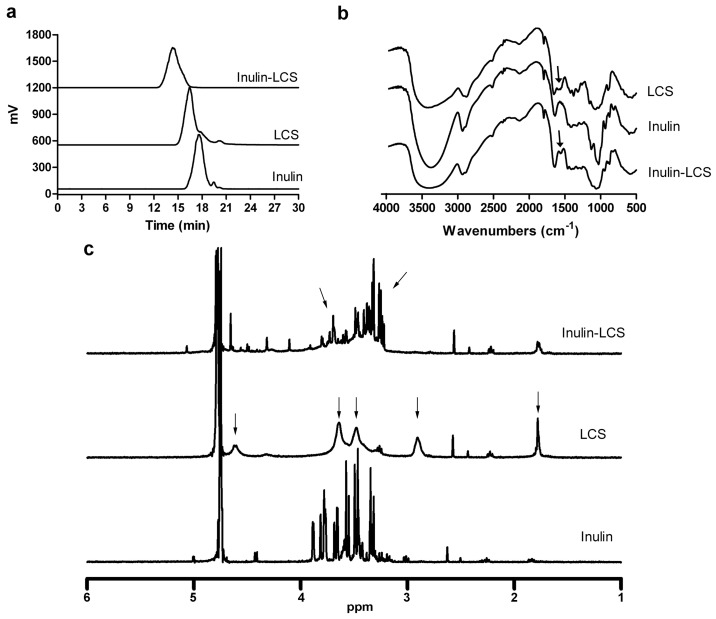
Physical characterizations of inulin–chitosan conjugate were measured by HPLC (**a**), FT-IR (**b**), and ^1^H NMR (**c**).

As measured by HPLC, inulin was eluted as a single and symmetric peak at 17.8 min, while free LCS was characterized with a retention time of 15.6 min (Figure 3a). After free LCS was linked to inulin by chemical conjugation, the peak shifted from 15.6 min to 14 min. The result indicated that the molecular weight of LCS was increased with inulin conjugation.

#### 2.2.2. IR Assay

FT-IR showed that there were three characteristic peaks for LCS at 3360 cm^−1^ for OH, 1380 cm^−1^ for C–O–C, and 1600 cm^−1^ for NH_2_ (Figure 3b). The oxygen bridge peaks of the skeletal vibrations involving the C–O stretching appeared between 1150 cm^−1^ and 1085 cm^−1^. As compared to LCS, the spectrum for inulin–LCS showed a weakened NH_2_-associated band near 1600 cm^−1^ for the N–H bending in the primary amine. Thus, the IR spectrum provided evidence for the reducing of the amino groups on the LCS chains by reaction with inulin.

#### 2.2.3. ^1^H NMR Assay

Next, the identity and structure of inulin, LCS and inulin–LCS conjugate were characterized by ^1^H NMR spectroscopy (Figure 3c, Figure S4). It can be observed that the sharp peak of deuterated water was at 4.7 ppm in all spectra. The protons of inulin were observed at 3.3–3.9 ppm, consistent with previous observations [23]. The ^1^H NMR spectrum of LCS exhibited the following characteristic signals: the broad peak at 4.6 ppm was attributed to the proton of carbon 1 of the glucosamine unit; two wide peaks at 3.6 and 3.5 ppm are due to the protons of carbon 3, 4, 5, and 6 of the glucosamine unit; the broad peak at 2.9 ppm was attributed to the proton of carbon 2 (connected to NH_2_) of the glucosamine unit; the peak at 1.8 ppm was assigned to the methyl protons of the *N*-acetyl groups, possibly due to the incomplete deacetylation of chitosan [7,24]. The spectrum of inulin–LCS showed the following characteristics: (1) the peak at 2.9 ppm in the spectrum of LCS disappeared in inulin–LCS; (2) there was a big difference at 3.2–3.9 ppm between the spectrum of inulin and inulin–LCS, whereby the peaks at 3.6–3.9 ppm in the spectrum of inulin became smaller while multiple peaks at 3.2–3.4 ppm appeared in the spectrum of inulin–LCS. These changes were presumably because some protons originally bonded to oxygen atom of inulin were connected to nitrogen atom of inulin–LCS, resulting from condensation and reductive amination between LCS and oxidized inulin. In addition, the peaks at 3.2–3.4 ppm in the spectrum of inulin–LCS were due to the protons of carbon 2 (connected to NH_2_) of the glucosamine unit originally assigned to 2.9 ppm in the spectrum of LCS, which shifted to higher field in the spectrum of inulin–LCS. Taken together, these results strongly suggested the formation of the inulin–LCS conjugate.

### 2.3. Biofilm Eradication

**Figure 4:**
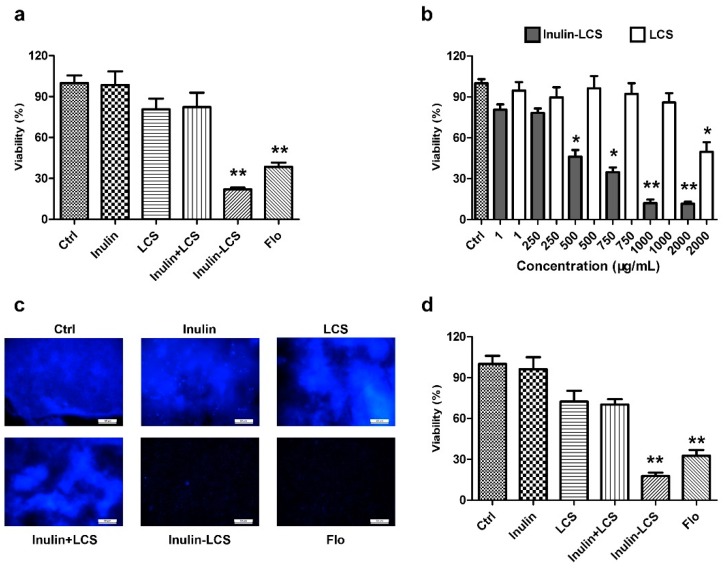
Eradicating (**a**–**c**) and inhibitory (**d**) activity of inulin–LCS on *S. aureus* biofilm was investigated. Florfenicol (250 μg/mL) was used as a positive control. The activity of biofilm eradication of inulin (1000 μg/mL), LCS (1000 μg/mL), their mixture (1000 + 1000 μg/mL) and inulin–LCS (1000 μg/mL) was determined by MTT assays (**a**). The concentration-dependency of inulin–LCS against biofilms was compared with LCS (**b**). After treatment with 1000 μg/mL of each sample, the biofilms were harvested for fluorescence image assay (**c**). Scale bar, 50 μm. The activity of biofilm inhibition of inulin (1000 μg/mL), LCS (1000 μg/mL), their mixture (1000 + 1000 μg/mL) and inulin–LCS (1000 μg/mL) was also measured by MTT assays (**d**). Data are represented as the means ± SD (*n* = 3). * *p* < 0.05 or ** *p* < 0.01, compared to the control or corresponding group.

The activity of biofilm eradication of inulin–LCS was evaluated, while equivalent amounts of inulin, LCS, or a mixture (inulin + LCS) were used for comparison. The results showed that 1 mg/mL of the inulin–LCS conjugate efficiently eliminated 78% mature *S. aureus* biofilm (Figure 4a). Neither inulin, chitosan, nor their physical mixture showed obvious activity at the same concentration (Figure 4a). As shown in Figure 4b, there was an obvious difference on biofilm eradication between inulin–LCS and LCS at concentrations of 500 μg/mL or higher (*p* < 0.05). These results were further confirmed by the results from fluorescence microscopical studies (Figure 4c). Furthermore, the anti-biofilm activity of inulin–chitosan was also compared with two common antibiotics, florfenicol and streptomycin. The conjugate (1000 μg/mL) showed similar activity on biofilm eradication with florfenicol at 500 μg/mL and streptomycin at 750 μg/mL (Supplementary Figure S3). All these results indicated that LCS modified with inulin (inulin–LCS) greatly improves the activity of LCS against mature *S. aureus* biofilm.

### 2.4. Biofilm Inhibition

Besides the activity of biofilm eradication, we also examined inulin–LCS on inhibiting the formation of the biofilm (Figure 4d). After treatment with inulin–LCS, the formation of biofilms was obviously impeded compared with the control group. While the inulin, chitosan, and their physical mixture showed no activity on biofilm inhibition. The results indicated that inulin–LCS could effectively inhibit the biofilm formation of *S. aureus* at 1000 μg/mL concentration.

### 2.5. Activity against Planktonic Bacteria

**Figure 5:**
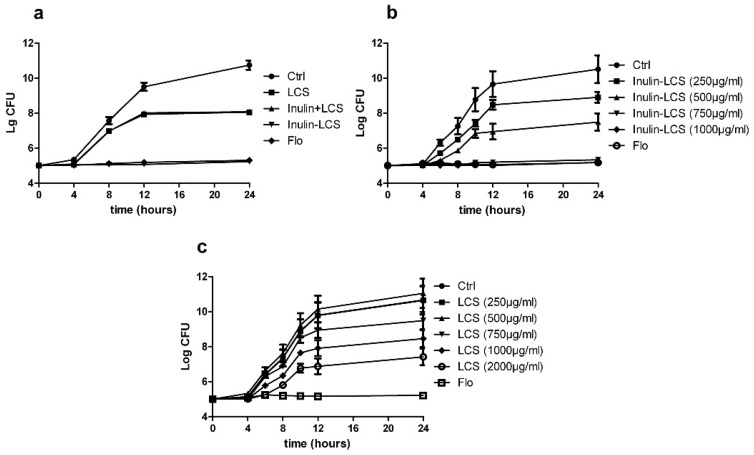
Inhibitory effects of inulin–LCS (1000 μg/mL) on planktonic *S. aureus* were investigated, while florfenicol (250 μg/mL) was used as a positive control (**a**). The growth of planktonic *S. aureus* under different concentrations of inulin–LCS and LCS were also investigated (**b**,**c**). Data are represented as the means ± SD (*n* = 3).

To investigate the effects of inulin–LCS on floating bacteria, shake-cultured *S. aureus* was treated with inulin, LCS, or inulin–LCS separately. Results showed that the growth of *S. aureus* was completely inhibited by inulin–LCS at 1000 μg/mL (Figure 5a) or even 750 μg/mL (Figure 5b). LCS could not fully inhibit bacteria growth even at 2000 μg/mL (Figure 5c). Inulin alone and the mixture of inulin and LCS did not show obvious inhibitory effects (Figure 5a). Thus, not just on biofilm, the antimicrobial activity of LCS on plankton was also greatly enhanced after the modification with inulin.

### 2.6. Broad Spectrum Anti-Biofilm Activity

**Figure 6:**
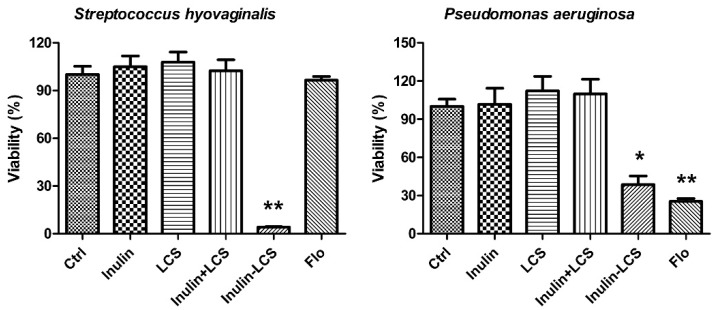
Broad spectrum anti-biofilm activity of inulin–LCS. Treatments of inulin–LCS on mature *Streptococcus hyovaginalis* and *Pseudomonas aeruginosa* biofilms were evaluated. Florfenicol (250 μg/mL) was used as a positive control. Data are represented as the means ± SD (*n* = 6). * *p* < 0.05 or ** *p* < 0.01, compared to the control.

Chitosan was reported to have broad spectrum antibacterial activity [25]. In this study, the anti-biofilm activity of inulin–LCS against both Gram-positive and Gram-negative bacteria was investigated (Figure 6). Although different to some extents, the results indicated that the inulin–LCS could efficiently eradicate mature biofilms of *Pseudomonas aeruginosa* (*p* < 0.05), a Gram-negative human pathogen. Interestingly, it effectively removed the biofilm of a *Streptococcus hyovaginalis* stain (*p* < 0.01), whose biofilm showed strong resistance to florfenicol (Figure 6). These results supported a broad spectrum anti-biofilm activity of the inulin–LCS conjugate.

### 2.7. Cellular Toxicity

**Figure 7:**
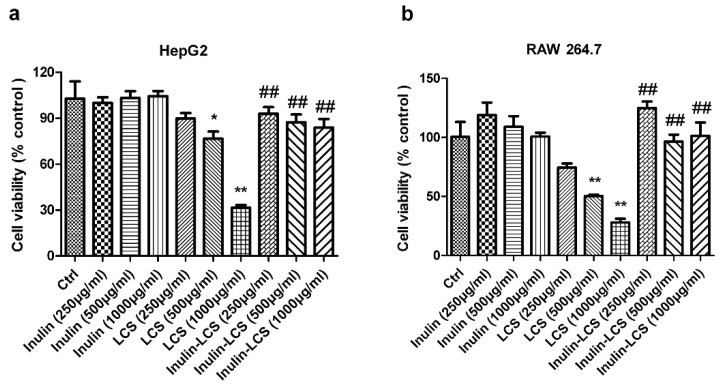
Effects of inulin, LCS and inulin–LCS on the viability of HepG2 (**a**) and RAW 264.7 cells (**b**). Data are represented as the means ± SD (*n* = 8). *p < 0.05 or ** *p* < 0.01, compared to the control group. ^##^ *p* < 0.01, compared to the LCS (1000 μg/mL) group.

To investigate possible side effects of the inulin–LCS conjugate, normal HepG2 and RAW 264.7 cells were chosen for cell viability analysis. Inulin–LCS (1 mg/mL) did not influence the growth of both mammalian cell strains (Figure 7). However, viability of HepG2 and RAW 264.7 cells was affected under LCS treatment when concentrations were greater than 500 μg/mL (*p* < 0.05 or *p* < 0.01). A significant difference (*p* < 0.01) between LCS treatment and that of inulin–LCS was shown, indicating that the cellular toxicity of LCS was dramatically decreased by the conjugation with inulin.

## 3. Discussion

*S. aureus* is a Gram-positive bacterium that typically colonizes the anterior nasopharynx and the surface of skin [26,27]. This bacterium is found in 30–50% of healthy individuals in the United States, and it was estimated that *S. aureus* had caused around $450 million losses in the past decade [28,29]. Many studies have found that *S. aureus* biofilm is a major cause for several types of infections including osteomyelitis, cystic fibrosis lung infection, implant-associated infections, and chronic diseases [6,30]. In fact, bacterial biofilm is tightly associated with the unsuccessful drug treatment of several chronic diseases and nosocomial infections, and biofilm-specific drugs are lacking. Thus, novel strategies to effectively combat biofilm-related infection are especially crucial. As a popular biological material, chitosan has showed antimicrobial activity against both planktonic bacteria and biofilm. In this study, we, for the first time, evaluated the anti-biofilm effect of the chitosan conjugates with inulin, a nontoxic water-soluble polysaccharide. Significantly enhanced activities of the inulin–chitosan conjugate against both planktonic *S. aureus* and its biofilm were observed compared with chitosan. Noticeably, it had broad spectrum anti-biofilm activity against Gram-positive and Gram-negative bacteria, and remarkably decreased cellular toxicity.

In most cases, chitosan derivatives were prepared by introducing small functional groups, including quaternization, phosphorylation, hydrophobization, or attachment of polyethyleneoxide [31,32,33]. In this study, we have developed a method of using one polysaccharide to modify chitosan to increase its activity. Inulin is a water-soluble polysaccharide and a widely used ingredient in food industries because of its health benefits and functional properties [34]. It was unsurprising to see that the water solubility of chitosan was greatly enhanced by conjugation with this highly water-soluble macromolecule (data not shown). Reduced cytotoxicity of the conjugate may also be due to the same reason. Thus, the conjugation of chitosan and inulin solved some drawbacks of chitosan, while preserving advantages of both polysaccharides at the same time. Research data have shown that inulin is associated with pathogen prevention, as evidenced by the enhanced killing of *Salmonella enteritidis*, and changed the composition or metabolic characteristics of the bacteria [20,21]. Thus, the promising anti-biofilm activity of inulin–LCS might be due to a synergistic effect of LCS and inulin. However, the molecular mechanism behind this activity requires further explorations.

Currently, antibiotics are still the most common tool for the treatment of acute microbial infection. However, they become less effective on biofilm-related infections, due to the drug-resistance of the biofilm [35]. Mechanisms involved in biofilm recalcitrance are depending on the class of antibiotic used and involved with different factors, including impaired antibiotic diffusion, drug indifference, expression of biofilm-specific genetic mechanisms, and the presence of persister cells [36]. Besides, some antibiotics such as azoles, polyenes, and echinocandins have been proved to have clinical drawbacks, due to their toxicity [37]. Thus, novel antibacterial candidates with enhanced efficacy against biofilm-related infections and less side effects are required to be developed. In the present study, a conjugate was developed based on two natural polysaccharides, inulin and chitosan, which had been commonly used in food production, biotechnology, and medicine. This novel conjugate displayed similar or even better effects on biofilm eradication and biofilm inhibition than that by florfenicol (250 μg/mL), a commonly used veterinary drug. In particular, it showed a suppressing inhibitory effect for florfenicol-resistant *Streptococcus hyovaginalis*. Furthermore, the anti-biofilm activity of inulin–chitosan (1000 μg/mL) was also compared with two commonly used antibiotics (florfenicol and streptomycin) at different concentrations (Supplementary Figure S3). In line with the above, the data showed that it has similar activity on biofilm eradication with florfenicol (500 μg/mL) and streptomycin (750 μg/mL). All these results suggested a promising practical value of inulin–chitosan on antimicrobial treatment. Effects of inulin–chitosan in animal models of bacterial infection and the molecular mechanism of the activity should be further elucidated in future studies.

Previous studies have demonstrated that there was a direct relationship between the antibacterial activity of chitosan and its structural characteristics, especially molecular weight (MW). It was reported that the antimicrobial activity increases with increase of the molecular weight (MW) of chitosan for Gram-positive *S. aureus* [25]. In addition, the influence of MW on biological activities was also studied by preparing oligosaccharides of chitosan (COS), which showed that COS have higher activity than the native polysaccharides [38]. In present study, the activities of COS, COS5k, and LCS against *S. aureus* bacteria/biofilm were systematically evaluated. On the contrary, our results indicate that chitosan is more effective against both planktonic bacteria and biofilm, while COS with a lower molecular weight seemed less effective. This inconsistency with previous studies might be due to the source of the chitosan and experimental settings. 

## 4. Materials and Methods 

### 4.1. Reagents and Bacterial Strains 

Chitosan oligosaccharide (COS) was prepared as previously described with the average molecular weight below 1 kDa [39]. COS5k (4–6 kDa) and low molecular weight chitosan (50–190 kDa, LCS) was purchased from Sigma (St. Louis, MO, USA). Inulin was prepared in our laboratory and the average molecular weight was determined to be 5 kDa by HPLC with 1 kDa, 5 kDa and 25 kDa dextran as standards (Supplementary Figure S1). 

*Staphylococcus aureus* (CGMCC 1.2910), *Pseudomonas aeruginosa* (PAO1) strains and *Streptococcus hyovaginalis* (CGMCC 1.10866) were purchased from China General Microbiological Culture Collection Center (CGMCC, Institute of Microbiology, CAS, Beijing, China), and the strains were cultured in tryptone soya broth (TSB) at 37 °C, MRS at 30 °C and Luria–Bertani (LB) medium at 30 °C, respectively.

### 4.2. Preparation of Inulin–Chitosan Conjugate

Inulin (4000 μg/mL, 5 mL) was oxidized by 20 mM NaIO_4_ in 20 mM acetate buffer (pH 5.8). The reaction was kept in dark for 30 min at room temperature, and was stopped by addition of excessive ethylene glycol, followed by extensive dialysis against water. The inulin–chitosan conjugate was obtained by incubation of chitosan (4 mg) with the oxidized inulin (20 mg) and NaCNBH_3_ (2 mg) in water at 4 °C overnight, after extensive dialysis against water, and lyophilized in vacuum. The method for quantitative analysis of chitosan was based on ninhydrin reaction, and the inulin/chitosan ratio (*w*/*w*) of inulin–chitosan was calculated to be 3.18.

### 4.3. Physicochemical Characterization

#### 4.3.1. IR Assay

Structural analysis of inulin–chitosan conjugate was performed by Fourier transform infrared spectroscopy (FT-IR). Samples were mixed thoroughly with potassium bromide in the mass ratio of 2:98 and punched to a tablet employing hydraulic press. The FT-IR data were recorded at a wave number range of 4000–400 cm^−1^ using a Thermo Scientific Nicolet iS10 FT-IR Spectrometer (Thermo Scientific, Waltham, MA, USA).

#### 4.3.2. HPLC Assay

The conjugate was measured by an Agilent 1260 for gel permeation chromatography (Agilent Technologies, Santa Clara, CA, USA) equipped with an evaporative light-scattering detector (ELSD). Chromatography was performed on two columns (TSKgel GMPWXL and TSKgel G2500PWXL, Tosoh Corporation, Tokyo, Japan) coupled in series with an overall dimension (7.8 mm × 600 mm), using 0.125 M NH_4_OAc aqueous solution (pH 4.5) as mobile phases at a flow rate of 1.0 mL/min with column temperature at 30 °C. The sample concentration was 1000 μg/mL. 

#### 4.3.3. ^1^H NMR Assay

The identity and structure of inulin, chitosan, and inulin–chitosan conjugate were analyzed by ^1^H NMR at 600 MHz. Freeze-dried inulin, chitosan, and inulin–chitosan conjugate were dissolved in deuterated water to a final concentration of 5000 μg/mL. The ^1^H NMR spectra were obtained by a Bruker NMR Spectrometer, Avance DRX 600 MHz, equipped with a 5 mm NMR probe (Bruker, Karlsruhe, Germany) at 25 ± 0.1 °C. MestReNova software was used to process the spectrum data.

### 4.4. Biofilm Eradication

For biofilm assay, 100 μL of TSB medium containing 1 × 10^7^ cfu/mL *S. aureus* was seeded into each well of a sterile flat-bottomed 96-well polystyrene micro-titer plate. The plate was covered and incubated at 37 °C for 24 h to allow cell attachment and biofilm formation. Then, the supernatant was removed, and the remainder was washed three times with PBS. After incubation at 37 °C with TSB containing compounds for different periods, as indicated, for 24 h, the culture supernatant was removed and washed with PBS. Then, 100 µL of MTT (5000 μg/mL) was added and incubated for another 4 h. Followed by the removal of MTT, the colored formazan was dissolved in 100 μL of DMSO. To determine the amount of biofilm, optical density (OD) values were measured at 490 nm using a Tecan Infinite M200 Pro Microplate Reader (Grodig, Austria). All test was performed in 6 replicates for each treatment. Each assay was performed with three biological triplicates.

### 4.5. Biofilm Inhibition

In this part, 100 μL of TSB medium containing 1 × 10^7^ cfu/mL *S. aureus* treated with different compounds was seeded into each well of a 96-well plate. After incubation at 37 °C for 24 h, the amount of biofilm was evaluated by MTT staining assay, as above. All tests were performed in 6 replicates for each treatment. Each assay was performed with three biological triplicates.

### 4.6. Fluorescence Microscopy Assay

For fluorescent staining, 3 mL TSB medium containing 1 × 10^7^ cfu/mL *S. aureus* were added to a sterile cell culture dish of 35 mm × 10 mm. After incubation at 37 °C for 24 h, the supernatant was removed and washed three times using PBS. The culture dish was incubated at 37 °C for 24 h in TSB supplemented with compounds for different periods, as indicated. Then, 4′,6-diamidino-2-phenylindole (DAPI) was added and incubated in dark for 0.5 h at 37 °C. After that, the culture dish was washed and fixed using a 4% paraformaldehyde solution for 0.5 h at 37 °C. The corresponding fluorescent images were taken by confocal laser scanning microscopy (CLSM) TCS SP5 (Leica, Germany).

### 4.7. Antibacterial Activity on Planktonic Bacteria

The antibacterial activity on planktonic bacteria was tested using the method developed for half maximal inhibitory concentration (IC_50_) determination. In brief, bacteria suspension (1 × 10^5^ cfu/mL in TSB) was inoculated into the bottles together with a serial concentration of inulin, LCS, and inulin–LCS respectively, and incubated at 37 °C with shaking incubator for 24 h. Then, the bacteria cells were spread on TSB plates, in order to test the growth rate and cell viability. All the tests were carried out in triplicate.

### 4.8. Cellular Toxicity Assay 

Human HepG2 cells and murine RAW 264.7 macrophage cells were obtained from the American Type Culture Collection (Manassas, VA, USA). The cells were grown in Dulbecco’s modified eagle medium (DMEM) containing 10% fetal bovine serum (FBS), 100 units/mL penicillin, and 100 µg/mL streptomycin, at 37 °C under a 5% CO_2_ atmosphere.

The cell toxicity of inulin–chitosan conjugate was analyzed by the MTT assay [40]. Briefly, HepG2 and RAW 264.7 cells were incubated in 96-well plates (3 × 10^3^ cells/well) with different concentrations of inulin, chitosan, and conjugate (250, 500, and 1000 μg/mL) for 24 h. The MTT assay was performed as above. Cell viability (%) was calculated as (absorbance of sample/absorbance of control) × 100.

### 4.9. Statistical Analysis

Data are presented as means ± SD. A two-tailed Student’s *t* test was performed for the comparison between two groups and one-way ANOVA for multiple group analysis. The *p* value < 0.05 or 0.01 was considered be significant. All data were analyzed using Statistical Product and Service Solutions (SPSS) 19.0 software (SPSS, USA).

## 5. Conclusions

In conclusion, conjugation of inulin significantly enhanced chitosan activity on biofilm eradication, biofilm inhibition, and anti-planktonic bacterial activity. Furthermore, it had broad-spectrum antimicrobial activity and markedly low cellular toxicity to mammalian cells. The characteristics of chitosan conjugation of inulin, including good solubility and nontoxic properties, mean that it is a good candidate for treatment against biofilm-related infections.

## Supplementary Materials

The following are available online at http://www.mdpi.com/1660-3397/16/5/151/s1, Figure S1: The average molecular weight of inulin was 5 kDa by HPLC using ELSD detection on a TSKgel G4000PWXL (Tosoh corporation) column (7.8 mm × 300 mm), with 1 kDa, 5 kDa and 25 kDa dextran as standards. Figure S2: Activities of different molecular weight chitosan (2000 μg/mL) against the *S. aureus* plankton were investigated. Florfenicol (250 μg/mL) was used as a positive control. Figure S3: The concentration-dependent inhibition of florfenicol (Flo) and streptomycin (Strep) against *S. aureus* biofilms was compared with inulin–LCS (1000 μg/mL). Data are represented as the means ± SD (*n* = 8). * *p* < 0.05 or ** *p* < 0.01, compared to the inulin–LCS group. Figure S4: The ^1^H NMR spectra of inulin, chitosan, and inulin–chitosan conjugate as followed. Figure S5: The fluorescence image of biofilms treated with 1000 μg/mL of each sample. Scale bar, 50 μm.
