# Supplementary material for: Conjugation of Inulin Improves Anti-Biofilm Activity of Chitosan
Source: Mar Drugs. 2018 May 4;16(5):151. doi: 10.3390/md16050151 (PMC5983282; doi:10.3390/md16050151)

Supplementary Information

Fig. 1. The inulin was obtained from the roots of *Helianthus tuberosus* as previously described method (*International Journal of Biological Macromolecules* **2003**, 33:135-140). It was isolated by water extraction and ethanol precipitation, followed by ion-exchange chromatography and gel filtration of the crude precipitate. The average molecular weight of inulin was 5 kDa by HPLC using ELSD detection on a TSKgel G4000PWXL (Tosoh corporation) column (7.8 mm× 300 mm), with 1 kDa, 5 kDa and 25 kDa dextran as standards.

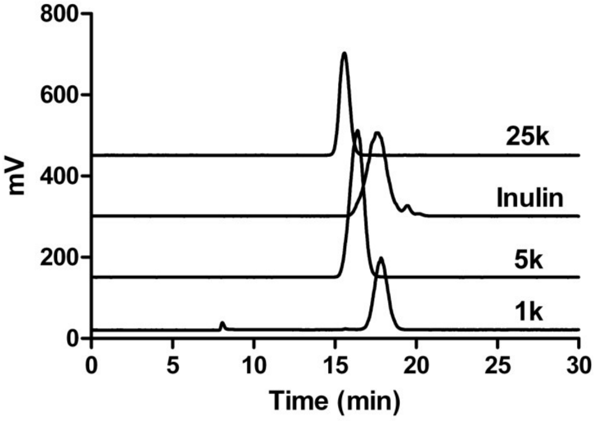

Fig. 2. Activities of different molecular weight chitosan (2000 μg/ml) against the *S. aureus* plankton were investigated. Florfenicol (250 μg/ml) was used as a positive control.

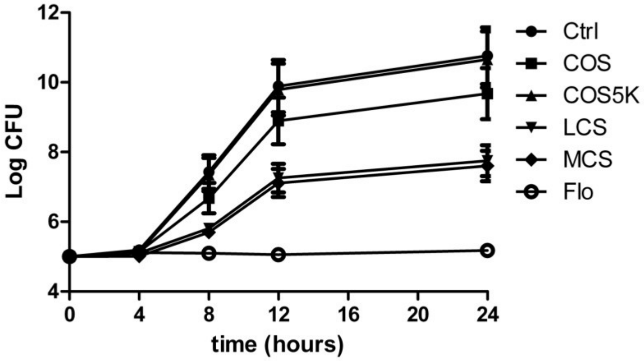

Fig. 3. The concentration-dependent inhibition of florfenicol (Flo) and streptomycin (Strep) against *S. aureus* biofilms was compared with Inulin-LCS (1000 μg/ml). Data are represented as the means ± SD (n=8). **P*<0.05 or ***P*<0.01, compared to the Inulin-LCS group.

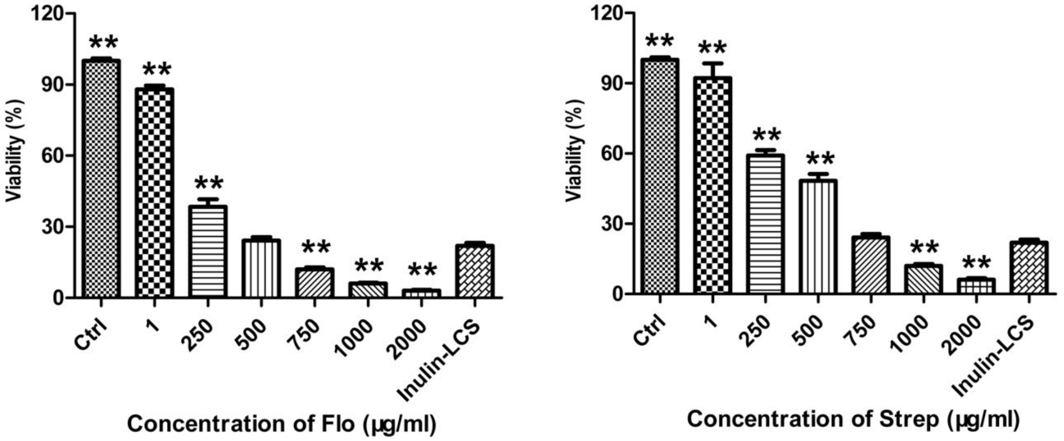

Fig. 4. The ^{1}H-NMR spectra of inulin, chitosan and the inulin-chitosan conjugate as followed.

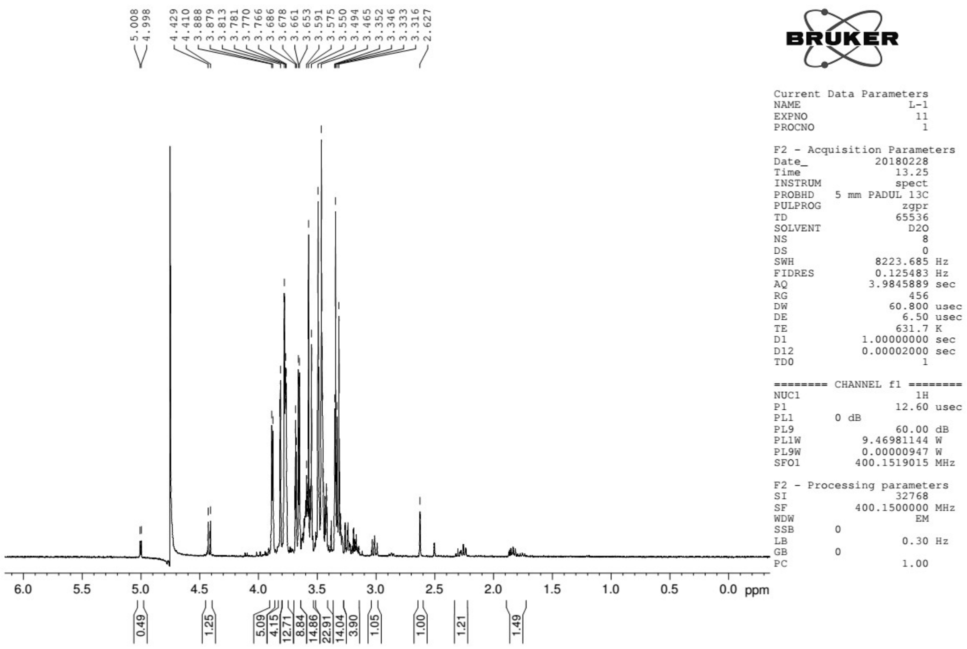

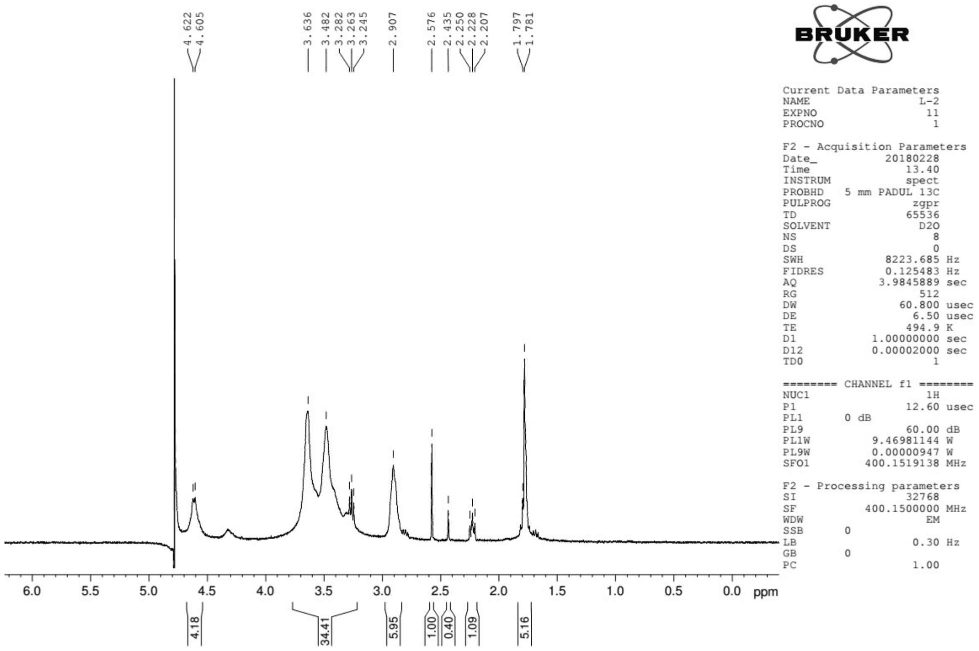
4.622
4.605
3.636
3.482
3.282
3.263
3.245
2.907
2.576
2.435
2.250
2.228
2.207
1.797
1.781
BRUKER
Current Data Parameters
NAME L-2
EXPNO 11
PROCNO 1
F2 - Acquisition Parameters
Date_ 20180228
Time 13.40
INSTRUM spect
PROBHD 5 mm PADUL 13C
PULPROG zgpr
TD 65536
SOLVENT D2O
NS 8
DS 0
SWH 8223.685 Hz
FIDRES 0.125483 Hz
AQ 3.9845889 sec
RG 512
DW 60.800 usec
DE 6.50 usec
TE 494.9 K
D1 1.00000000 sec
D12 0.00002000 sec
TD0 1
======== CHANNEL f1 ========
NUC1 1H
P1 12.60 usec
PL1 0 dB
PL9 60.00 dB
PL1W 9.46981144 W
PL9W 0.00000947 W
SFO1 400.1519138 MHz
F2 - Processing parameters
SI 32768
SF 400.1500000 MHz
WDW EM
SSB 0
LB 0.30 Hz
GB 0
PC 1.00
6.0
5.5
5.0
4.5
4.0
3.5
3.0
2.5
2.0
1.5
1.0
0.5
0.0
ppm
4.18
34.41
5.95
1.00
0.40
1.09
5.16

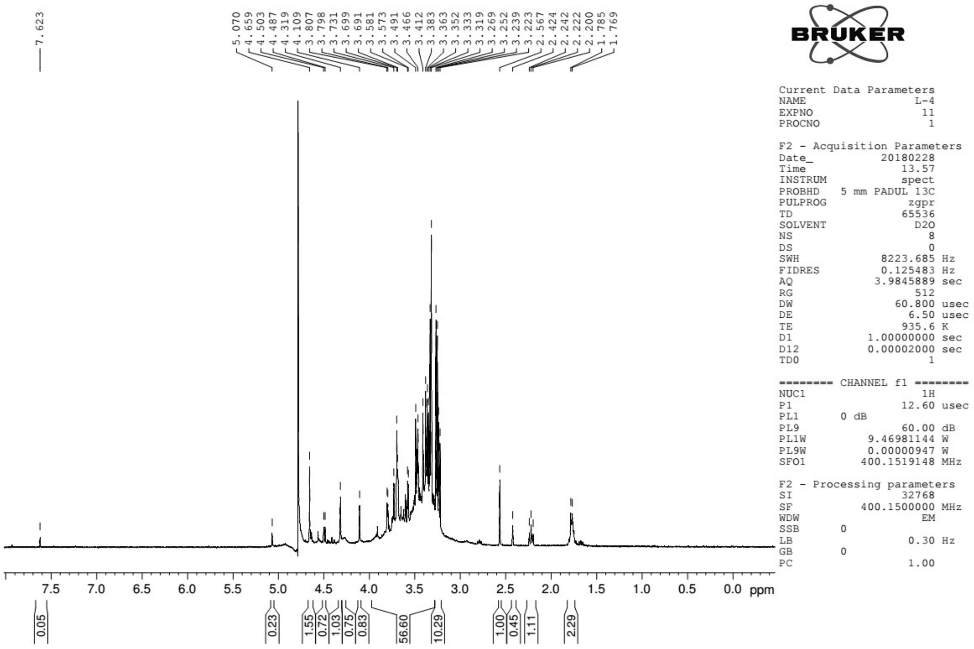
7.623
5.070
4.659
4.503
4.487
4.319
4.109
3.807
3.798
3.731
3.699
3.691
3.581
3.573
3.491
3.466
3.412
3.383
3.363
3.352
3.333
3.319
3.269
3.252
3.239
3.223
2.567
2.424
2.242
2.222
2.200
1.785
1.769
BRUKER
Current Data Parameters
NAME L-4
EXPNO 11
PROCNO 1
F2 - Acquisition Parameters
Date_ 20180228
Time 13.57
INSTRUM spect
PROBHD 5 mm PADUL 13C
PULPROG zgpr
TD 65536
SOLVENT D2O
NS 8
DS 0
SWH 8223.685 Hz
FIDRES 0.125483 Hz
AQ 3.9845889 sec
RG 512
DW 60.800 usec
DE 6.50 usec
TE 935.6 K
D1 1.00000000 sec
D12 0.00002000 sec
TD0 1
======== CHANNEL f1 ========
NUC1 1H
P1 12.60 usec
PL1 0 dB
PL9 60.00 dB
PL1W 9.46981144 W
PL9W 0.00000947 W
SFO1 400.1519148 MHz
F2 - Processing parameters
SI 32768
SF 400.1500000 MHz
WDW EM
SSB 0
LB 0.30 Hz
GB 0
PC 1.00
7.5
7.0
6.5
6.0
5.5
5.0
4.5
4.0
3.5
3.0
2.5
2.0
1.5
1.0
0.5
0.0
ppm
0.05
0.23
1.55
0.72
1.03
0.75
0.83
56.60
10.29
1.00
0.45
1.11
2.29

Fig. 5. The fluorescence image of *S. aureus* biofilms treated with 1 mg/ml of each sample. Scale bar, 50 μm.

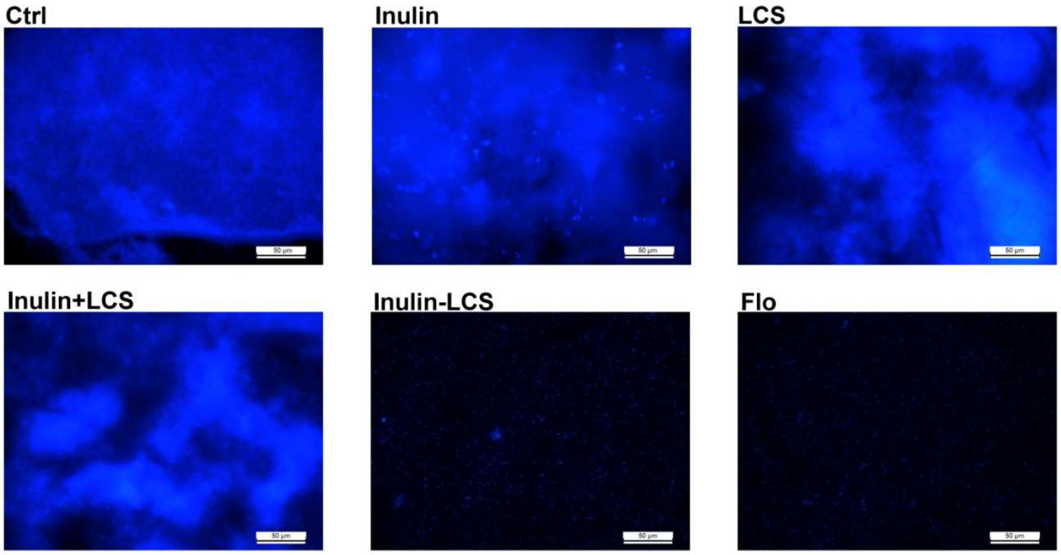

Supplement: Supplementary file 1 [file marinedrugs-16-00151-s001.zip › marinedrugs-296240-SI.pdf]
